# Synthesis and characterization of comb-shaped copolymer as a filtration reducer and comparison with counterparts

**DOI:** 10.1039/c7ra13255g

**Published:** 2018-03-22

**Authors:** Lu Liu, Xiaolin Pu, Huaizhi Tao, Qing Deng, Ang Luo

**Affiliations:** State Key Laboratory of Oil and Gas Reservoir Geology and Exploitation, Southwest Petroleum University Chengdu 610500 China puxiaolin@vip.sina.com; Drilling & Production Technology Research Institute, CNPC Sichuan Petroleum Guanghan Sichuan 618300 China

## Abstract

A comb-shaped copolymer of 2-acrylamide-2-methyl propane sulfonic acid (AMPS), allyl polyoxyethylene ether (APEG), *N*-vinyl-2-pyrrolidone (NVP) and sodium styrene sulfonate (SSS) was synthesized by free-radical polymerization. The structure of the comb-shaped copolymer was characterized by Fourier transform infrared (FTIR) spectroscopy, and its molecular weight was determined by gel permeation chromatography (GPC). FTIR measurements and environmental scanning electron microscopy (ESEM) analysis were used to characterize the working mechanism of different filtrate loss reducers. Thermogravimetry and differential scanning calorimetry (TG-DSC) results showed that thermal degradation of the copolymer was significant only after 295.24 °C. The comb-shaped copolymer helped reduce filtration, while maintaining the rheological properties of the drilling fluid at high temperature and high salinity conditions as long PEG chains sterically stabilized colloids by protruding into the suspension. The filtration control of the comb-shaped copolymer was comparable to that of the sulfonated phenolic resin (SMP) mixture and outperformed AM/AMPS/NVP/SSS (NS-1) and polymeric product PAC in terms of high-temperature resistance and rheological advantages. The morphology of the comb-shaped copolymer was found with a compact 3-D film structure due to the intramolecular and intermolecular association by hydrogen bonding in the side chains. Small curly debris at high temperature and salinity remained capable of filtration control. The NS-1 had a lower temperature resistance, as large areas of flaky films thermally degraded into a small chain structure at 180 °C. Only separated filiform and coarse lines were found in PAC with a linear structure that makes the drilling fluid more viscous. Compact and structured films were formed with the SMP mixture at high temperature and salinity.

## Abstract 2

Comb-shaped copolymer as filtration reducer for high temperature and high salinity.

## Graphical abstract

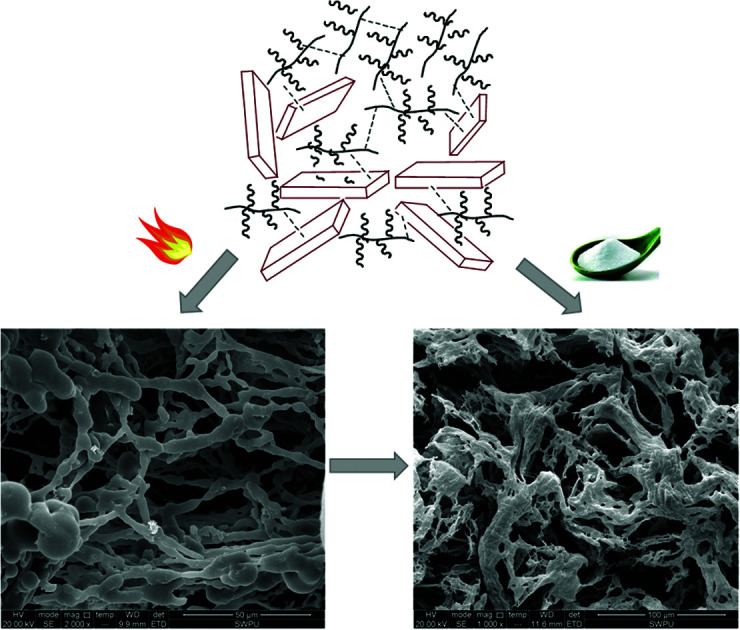

## 1 Introduction

Drilling fluids are essential for the oil, gas and geothermal drilling industry, as they perform functions including controlling formation pressure, minimizing formation damage, transporting rock cuttings to the surface and minimizing filtration into permeable formations by forming a high-quality filter cake.^1^ When drilling into a deep environment, high temperatures are often encountered, which adversely affect the performance of drilling fluids. In particular, bentonite suspensions may lose filtration control, resulting in severe formation damage and change in the rheological properties of drilling fluids.^2,3^ Such situations may get worse in the case of zones containing a high concentration of salts. To address such problems, filtrate loss reducers are often used to control the filtration into formation and stabilize the bentonite dispersion in a high-temperature and high-salinity environment.^4–7^

Generally, sulfonated phenolic resin (SMP) compounds are the most frequently used products to control filtration volume. However, their usage may cause deterioration of the rheological properties of drilling fluids once excessive cross-linked behavior occurs at high temperature. Further, such a cross-linking structure in SMP blends is often difficult to control and handle. Alternatively, a great diversity of polymeric additives functioning as filtrate loss reducers have been introduced, and their use often increases the viscosity of the drilling fluid despite the high-temperature resistance.^8–10^ More often, their salinity tolerance is overlooked. Therefore, it is a great challenge to prepare polymeric filtrate loss reducers and maintain a bentonite dispersion for a high-temperature and high-salinity environment.

Drilling fluids are primarily water-based bentonite suspensions and their colloidal stability is maintained by dispersive agents. According to colloidal science,^11,12^ the adsorption of a dispersive agent on the bentonite clay particles' surface makes them available for electrostatic and/or steric barriers against flocculation.^13–15^ Electrostatic repulsion is often achieved by adsorbing a multitude of charged groups on the chains to increase zeta potentials. However, electrostatic repulsion itself is extremely sensitive to the presence of the electrolyte in the medium, because of which the thickness of the double layer is reduced and the electrostatic potential barrier is lowered.^16^ An alternative approach is to utilize brush-like or comb-shaped copolymers bearing certain anchoring groups and a long hydrophilic segment for steric stabilization. Anchoring groups facilitate the strong adsorption of copolymers on the surface of clay particles, whereas long hydrophilic chains protrude from the particle surface. Therefore, the adsorbed layers are increased to a level that enables effective steric stabilization.^17^ Steric stabilization is generally less sensitive to the electrolyte and works well for a high-salinity environment.

Comb-shaped copolymers have superior colloidal maintenance ability in relation to regular linear copolymers at high temperature and salinity and have been widely used for stabilizing emulsions and suspensions in biological medicine, petroleum, cement and plastics industries.^18^ Numerous reports have documented that comb-shaped structures bearing a sulfonic group as the anchoring group and PEG chains as the hydrophilic segment that protrudes into aqueous phase impart steric repulsion.^17,19,20^ However, little work has been related to comb-shaped copolymers for the processing of bentonite dispersions,^21–25^ and their application as filtrate loss additives for high temperature and high salinity environments in a petroleum field have not yet been reported.

The aim of this work is to develop a comb-shaped copolymer as a filtrate loss reducer for high-temperature and high-salinity conditions. With this in mind, the free-radical copolymerization of AMPS, APEG, NVP and SSS monomers is conducted. Nonionic amide moieties serve as anchoring group, allowing the adsorption onto the clay particle surface. Long PEG chains in the comb-shaped structure can protrude into the clay suspension medium and sterically hinder colloidal aggregation at high temperature and salinity. Ionizable groups such as the sulfonic group increase the hydration shell around clay particles and improve salinity tolerance. Multiple ring structures are incorporated to help enhance the rigidity of the main chains and improve the temperature tolerance.

## 2 Materials and methods

### 2.1 Materials

2-Acrylamide-2-methyl propane sulfonic acid (AMPS) [industrial grade (IR)], allyl polyoxyethylene ether (APEG) (IR), ammonium persulfate (APS) [analytical grade (AR)] and sodium bisulfate (NaHSO_3_) (AR) were purchased from Chengdu Kelong Reagent Plant, Chengdu, Sichuan, P. R. China. Sodium styrene sulfonate (SSS) (IR) was obtained from Shanghai Bangcheng Chemical CO., LTD. Shanghai, P. R. China. *N*-Vinyl-2-pyrrolidone (NVP) (AR) was obtained from Sahn Chemical Technology Company, Shanghai, P. R. China. Bentonite was purchased from Xinjiang Xia Zi Jie Limited Company, Karamay, Sinkiang, P. R. China. PAC (IR), SMP (IR) and SMC (IR) were obtained from Gaoke Chemicals CO., LTD. Renqiu, Henan, P. R. China. Purification of AMPS was required beforehand. AMPS powders were dissolved in excess anhydrous methanol for 30 min and recrystallized under N_2_ at 50 °C.

### 2.2 Synthesis of comb-shaped copolymer

**Scheme 1:**
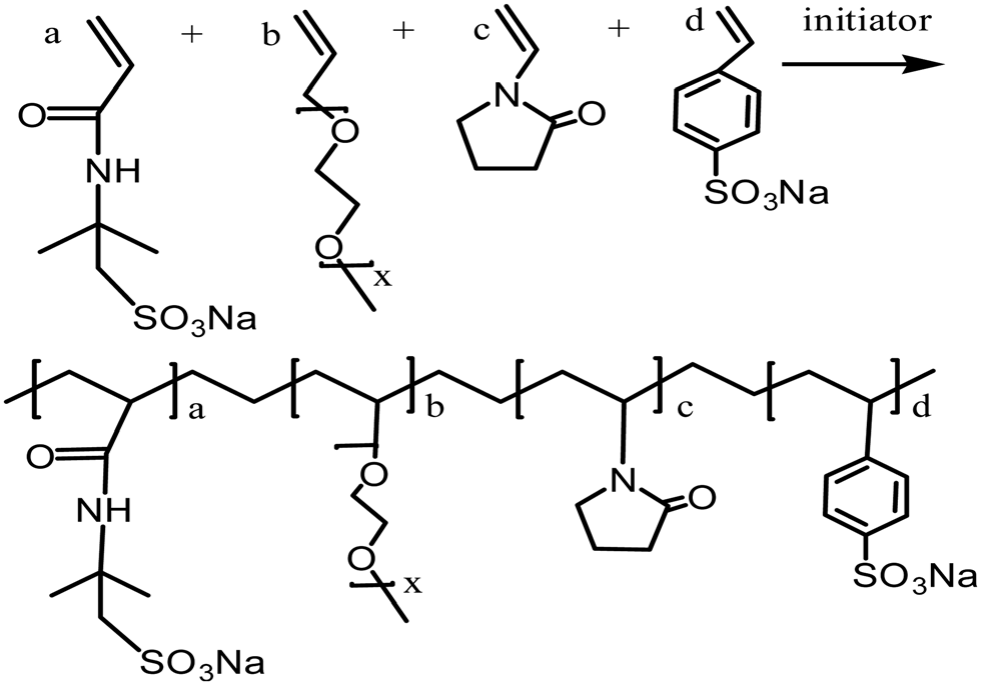
Proposed copolymerization of monomers AMPS, APEG, NVP and SSS.

The comb-shaped copolymer was prepared by aqueous free-radical copolymerization of four monomers: AMPS, APEG, NVP and SSS. The copolymerization was carried out in a homogeneous distilled water (DI water) system and was constantly protected by N_2_. In a typical procedure, predetermined AMPS was dissolved in DI water contained in a reaction flask and was neutralized to pH of 7–8 by adding 1 M NaOH solution. Then, NVP, SSS and APEG were added to the solution within 10 min, in sequence. The molar ratio of the monomer was AMPS : APEG : NVP : SSS = 7 : 3 : 2 : 2. The total monomer concentration was 13.5% based on the total weight of the reaction monomers. The reactive system was heated up to a reaction temperature of 50 °C and then initiators (APS and NaHSO_3_, each at 0.25% mass fraction) were added. The reaction was stirred for 5 hours at reaction temperature. While being cooled to room temperature, the reaction mixture was precipitated in anhydrous ethanol several times without any further purification. The obtained white powder was dried under vacuum at 50 °C for 6 h. The proposed chemical structure of the comb-shaped copolymer is shown in Scheme 1.

### 2.3 Characterization of comb-shaped copolymer

Chemical structure of the synthesized copolymer was determined by an FTIR spectrometer. The dried copolymer specimen was grinded with KBr and pressed into a circular sheet. The FTIR spectra of the specimens were recorded on a Fourier transform infrared spectrometer (NICOLET 6700, Thermo Scientific Co., USA) over the wave number range of 400 cm^−1^ to 4000 cm^−1^.

Molecular weight of the synthesized copolymer was recorded using gel permeation chromatography (GPC, Alliance e2695, Waters, USA) at 35 °C.

The thermal stability of the copolymer was measured on a thermal analyzer (TGA/DSC, 1/1100 L, Mettler Toledo Co., Switzerland) in N_2_ atmosphere. The heating rate was 10 °C min^−1^, and the temperature ranged from 50 °C to 800 °C.

The self-assembly image of each specimen in the aqueous solution was characterized using an environmental scanning electron microscope (ESEM, Quanta 450, FEI, USA). The filtrate loss reducer was dissolved in DI water (1 wt%) and then a small spoon (about 0.5 g) of hydrated Na-MMT dispersion (4 wt%) was added. The liquid mixture was added into a sample hold, freeze-dried and coated with a thin layer of gold for observation.

### 2.4 Performance measurement of copolymer-loaded drilling fluid

Performance observations of the drilling fluid were carried out according to American Petroleum Institute (API) specifications and Chinese SY/T5621-31. The API filtration loss (FL_API_) of the drilling fluid was tested on a medium-pressure filtration apparatus (SD3, Qingdao Haixin Optical Co., China). The aging tests were performed in a frequency conversion rolling oven (BRGL7, Qingdao Tongchun Petroleum Instrument Co., China) at a set temperature for 16 h, and then cooled to room temperature for testing. The filtration volume was collected using standard Fann filter papers (Fann Instrument Co., Houston, TX, USA) by applying a pressure of 0.69 ± 0.03 MPa at room temperature. The HTHP filtration volume (FL_HTHP_) was measured on a high-temperature and high-pressure filtration apparatus (GGS42, Qingdao Jiaonan Tongchun Machinery Petroleum instrument, China) by applying a pressure of 4.2 ± 0.03 MPa and at the given temperature.

The rheological parameters apparent viscosity (AV), plastic viscosity (PV) and yield point (YP) were derived from the rotational speeds at 600 rpm and 300 rpm on a six-speed rotational viscometer (ZNN-D6, Qingdao Tongchun Petroleum Instrument Co., China) at room temperature (25 °C) and atmospheric pressure (0.1 MPa). Rheological parameters were calculated as follows:
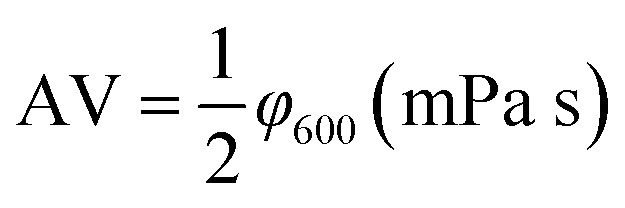
PV = *φ*__600__ − *φ*__300__ (mPa s)
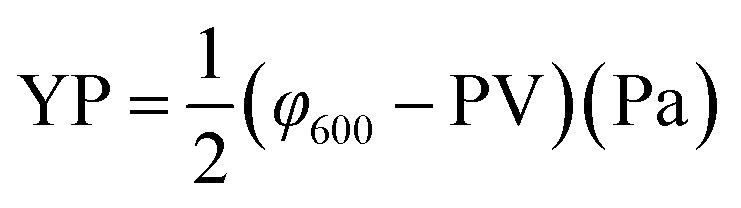


The terms *φ*_600_ and *φ*_300_ are dial readings on the viscometer at the rotating speed of 600 rpm and 300 rpm, respectively. To reach steady conditions, the fluids were stirred for 10 min prior to the rheological measurements.

## 3 Results and discussion

### 3.1 Composition of copolymer

**Fig. 1:**
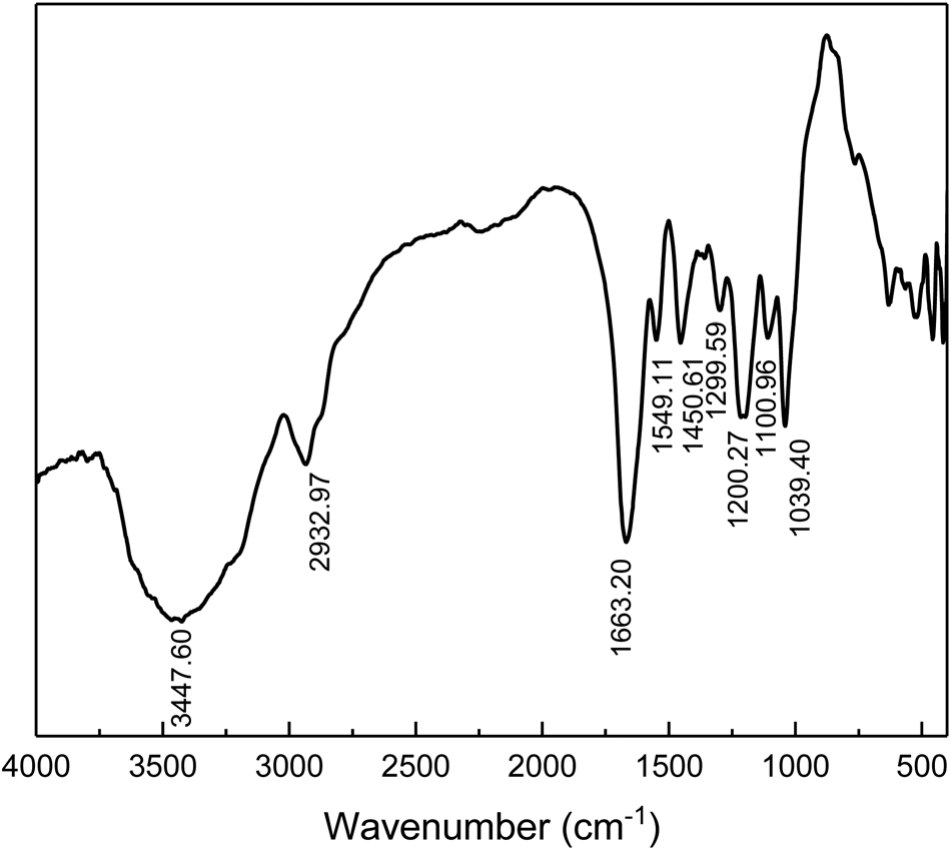
FTIR spectra of comb-shaped copolymer.

The chemical structure of the prepared comb-shaped copolymer was determined by FTIR spectroscopy, as shown in Fig. 1.

As indicated in Fig. 1, four characteristic bands of AMPS, APEG, SSS and NVP monomers are presented in the spectrum. The absorption peaks at 3447.60 cm^−1^ (N–H stretching band) and 1549.11 cm^−1^ (N–H bending band and C–N stretching band) correspond to the AMPS. The absorption peak at 1663.20 cm^−1^ (C=O group) corresponds to amide. The intense peak at about 1039.40 cm^−1^ (SO_3_^−^ stretching band) is attributed to AMPS and SSS. The absorption peak at 1450.61 cm^−1^ (benzene stretching band) corresponds to SSS. The absorption peak at 1299.30 cm^−1^ (C–N vibrating band) is assigned to NVP. The absorption peak at 1200.27 cm^−1^ (allyl polyethylene glycol) is attributed to APEG. The presence of these typical absorption bands in the FTIR spectrum indicates the occurrence of copolymerization of the fed monomers.

The molecular weight and distribution of the comb-shaped copolymer was measured in GPC, as shown Table 1.

**Table 1:** Molecular weight and its distribution for the synthesized copolymer

Sample	Elution volume (μL)	Retention time (min)	*M*_n_	*M*_w_	*M*_p_	*M*_*z*_	*M*_*z*+1_	PDI (*M*_w_/*M*_n_)
Copolymer	50.00	90.00	328 092	577 299	691 392	764 054	876 792	1.814

As shown in Table 1, the number average molecular weight (*M*_n_) is 328 092, the weight average weight (*M*_w_) is 577 299 and polydispersity index (PDI) of the specimen is 1.814. The relatively medium molecular weight is suitable for use as a filtrate loss reducer. The narrow distribution suggests a concentrated distribution of copolymer weight. Combined with the FTIR spectra, it can be concluded that a comb-shaped copolymer is successfully synthesized.

**Fig. 2:**
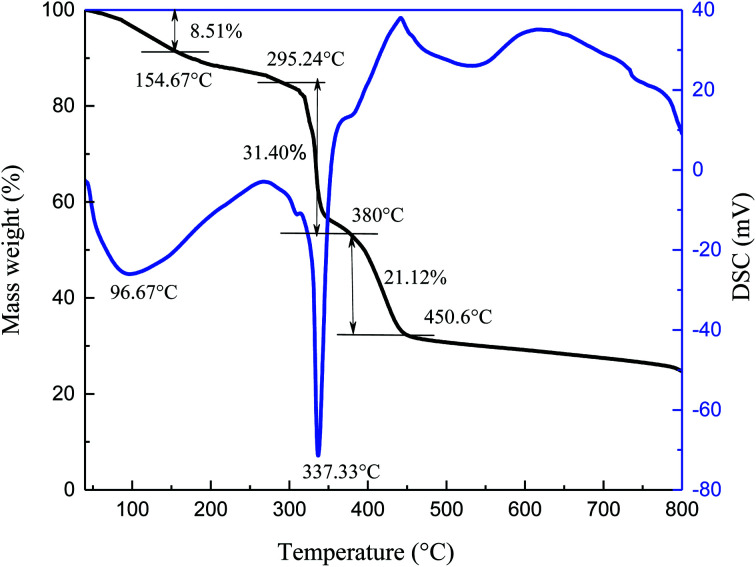
TG-DSC analysis of comb-shaped copolymer.

The thermal stability of specimen was observed by TG-DSC in N_2_ atmosphere, and the resulting curves are shown in Fig. 2.

The process of thermal decomposition of the synthesized copolymer includes three stages (Fig. 2). From the TG curve, the first stage is from 40 °C to 154.67 °C and accounts for about 8.51% of the weight loss. The loss rate is relatively slow during this stage and the largest mass loss occurs at 96.67 °C. It is mostly due to the evaporation of volatile substances and adsorbed water upon heating, as well as the decomposition of small amounts of unreacted residues. A drastic weight loss begins at 295.24 °C and ends at about 380 °C, corresponding to about 31.40% weight loss. A wave hollow appears in the DSC curve at 337.33 °C, which is related to the decomposition of functional groups in the synthesized copolymer. The third stage in the TG curve is from 380 °C to 450.6 °C, during which the mass decreases by 21.12%. The mass loss is caused by thermal degradation of the C–C bonds in the main chains at high temperature. Xin^26^ reported that the AMPS/AA/DMAA/SA copolymer showed no significant mass loss below 289 °C. Bai^27^ found that the thermal degradation of the synthesized AM/AMPS/DMDAAC/SSS copolymer was not obvious before 273.2 °C. It can be concluded that the thermal degradation of the copolymer is significant only after 295.24 °C and the synthesized copolymer itself exhibits good temperature resistance.

### 3.2 Performance of copolymer-laden drilling fluid

Flow behavior of the bentonite suspension system consisting of various agents is crucial for efficient drilling operations.^28^ The filtration of the drilling fluid through permeable zones must be controlled to avoid formation damage and reduction in the final oil and gas production. Therefore, it is essential to investigate the impact of different parameters on the drilling fluid properties.

#### 3.2.1 Rheological properties of copolymer-loaded drilling fluid

A series of copolymer-loaded drilling fluids were tested in fresh water, salt water and calcium drilling fluid at different concentrations. The rheological parameters as a function of copolymer concentration (Table 2), salt content (Table 3) and calcium content (Table 4) measured before and after aging process at 200 °C for 16 h are shown.

**Table 2:** Rheological properties as a function of copolymer concentration before and after aging at 200 °C

Test samples	AV (mPa s)		PV (mPa s)		YP (Pa)		YP/PV (Pa (mPa s)^−1^)	
	Before aging	After aging	Before aging	After aging	Before aging	After aging	Before aging	After aging
Base fluid	9.0	6.0	6	5	3.0	1.0	0.50	0.20
0.5	16.5	9.5	11	7	5.5	2.5	0.50	0.36
1	17.0	12.5	12	9	5.0	3.5	0.42	0.39
1.5	18.5	13.0	13	10	5.5	3.0	0.42	0.30
2	22.0	16.5	15	12	7.0	4.5	0.47	0.37

**Table 3:** Effect of salt on the rheological properties of comb-shaped copolymer before and after aging at 180 °C

NaCl concentration (wt%)	AV (mPa s)		PV (mPa s)		YP (Pa)		YP/PV (Pa (mPa s)^−1^)	
	Before aging	After aging	Before aging	After aging	Before aging	After aging	Before aging	After aging
5	20.5	16.5	16	14	3.5	2.5	0.21	0.17
10	18.5	15.5	15	13	3.5	2.5	0.23	0.19
15	17.5	13.0	13	11	4.5	2.0	0.35	0.18
20	16.0	10.5	12	9	4.0	1.5	0.33	0.16

**Table 4:** Effect of calcium on the rheological properties of copolymer before and after aging at 180 °C

CaCl_2_ concentration (wt%)	AV (mPa s)		PV (mPa s)		YP (Pa)		YP/PV (Pa (mPa s)^−1^)	
	Before aging	After aging	Before aging	After aging	Before aging	After aging	Before aging	After aging
0.25	24.0	20.0	18	15	6.0	5.0	0.33	0.33
0.5	21.5	18.5	16	14	5.5	3.5	0.34	0.25
0.75	18.5	15.5	14	13	4.5	2.5	0.32	0.19
1	17.5	11.5	13	10	4.5	1.5	0.34	0.15

Table 2 shows the effect of copolymer concentration on its rheological properties in fresh water-based drilling fluids. With increasing comb-shaped copolymer content into the system, the AV values increase with copolymer concentration. The comb-shaped copolymer strengthens the interlinked structure between the clay particles and copolymers by introducing more steric hindrance of the long side chains, which hinders the flow of the fluid. However, the molecular weight of the comb-shaped copolymer is modest and does not increase the viscosity of the drilling fluid sharply.

The viscosity after aging is lower than that before aging. In the bentonite suspension, there exists a competition between aggregation and dispersion of clay particles.^29^ High temperature increases thermal motion of the clay particles and results in a higher rate of collision; thus, clay particles are liable to aggregate at high temperature. However, the comb-shaped copolymer helps maintain bentonite suspension stability through high degree of steric hindrance imparted by long side chains and electrostatic repulsion between ionic groups. The aggregation degree of the clay particles is, therefore, reduced and hindered by the comb-shaped structure. The bentonite suspension is kept well dispersed and maintains a suitable viscosity. Moreover, a higher content of the comb-shaped copolymer in the system is conducive to maintaining viscosity at high temperature, as more long side chains are present in the suspension, promoting colloidal stability.

The YP/PV value is an indication of shear thinning viscosity of drilling fluids. High viscosity of the fluids at lower shear rates is required to suspend and transport drilled cuttings. The low viscosity of drilling fluids at higher shear rates enables the fluids to be pumped into the drilled hole efficiently. According to practical experience and flow behavior calculation, the recommended YP/PV value is between 0.36 and 0.48. In this case, the YP/PV value of the drilling fluids containing the comb-shaped copolymer ranges from 0.37 to 0.48 when the concentration exceeds 1 wt%. This range is not too large to cause pumping difficulty and not too small to affect the carrying capacity.

Harsh environment is inevitably encountered when drilling into gypsum zones or zones containing high-salinity formation water. Therefore, it requires drilling fluid agents with excellent salinity resistance and helps maintain the rheological properties of the system. We carried out salt and calcium contamination tests of the drilling fluid loaded with 2 wt% comb-shaped copolymer. The rheological properties are shown in Tables 3 and 4.

According to Tables 3 and 4, the salinity resistance of the comb-shaped copolymer was tested upon addition of various contents of salt and calcium chloride. It is found that the addition of salt and calcium can decrease the viscosity of the drilling fluid. The more the salinity of the system, the lower the viscosity of the drilling fluid before and after aging. This mainly results from salt-screening effect,^10,30^ which could decrease electrical double layer on the clay particles, which has an adverse effect on the colloidal dispersion.^31^ However, the trend of decreasing viscosity in the saline drilling fluid is modest because of the comb-shaped structure. Long PEG chains not only sterically stabilize the colloids by intrusion into the suspension media and altering the interconnectivity between the clay particles and copolymers,^32^ but also effectively screen the effect of Ca^2+^ onto the clay particles and protect the backbones from aggregation.^33^ The improved colloidal stability retains the rheological performance even at high temperature and high salinity. Furthermore, the addition of salt could decrease the electrical repulsion between the negative groups on the copolymer and inherently negative point on the clay particles, so that the adsorbing capacity of the copolymers onto clay particles is increased due to electrical attraction.^34^ Additionally, ionic group SO_3_^−^ is less sensitive to salinity difference and helps increase the hydration shell on clay particles. Therefore, it is concluded that the comb-shaped copolymer maintains suitable rheological properties of the drilling fluid at high salinity.

#### 3.2.2 Filtrate properties of copolymer-loaded drilling fluid

**Fig. 3:**
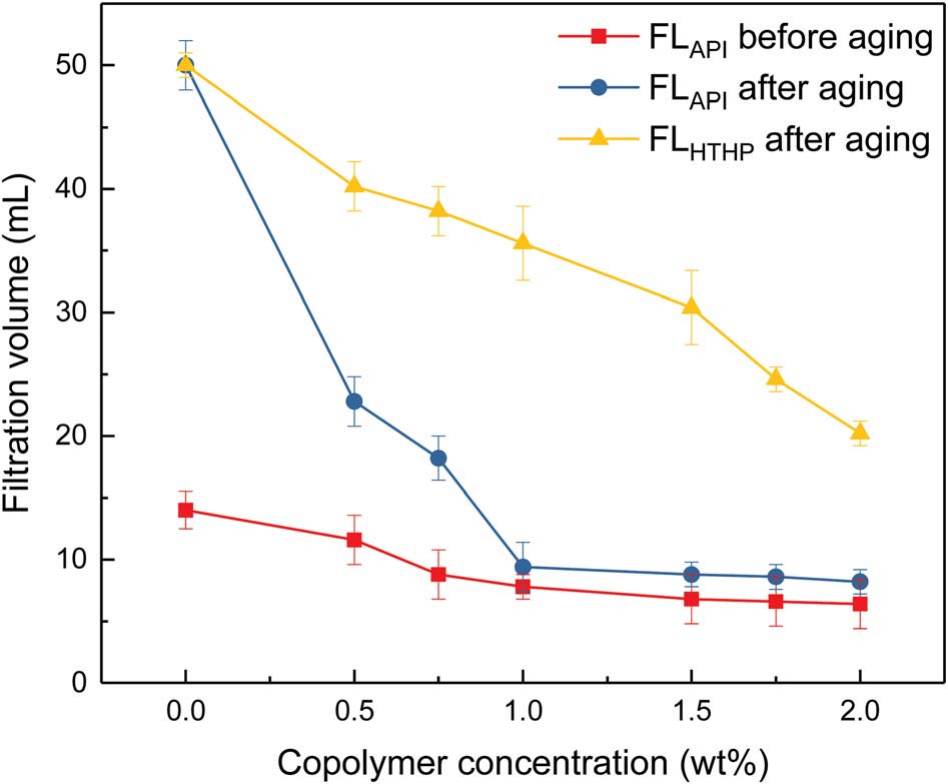
Filtrate loss volume of drilling fluid at varied copolymer concentrations. (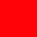) API filtration loss volume before aging. (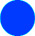) API filtration loss volume after aging at 200 °C. (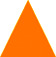) HTHP filtrate loss volume after aging at 200 °C.

The invasion of the liquid phase of the drilling fluid into rock formation must be controlled to avoid damage to the productive zones. The filtration volume of drilling fluids loaded with copolymers at varied concentrations were recorded and the results are shown in Fig. 3.

It can be seen from Fig. 3 that the filtrate volumes decrease with increasing copolymer concentration. The filtrate loss volume without the copolymer is above 50 mL after aging, showing severe mud losses. When the copolymer dosage increases to 1 wt%, the API loss volumes are below 10 mL before and after the 200 °C aging process. Further increasing the copolymer concentration up to 2 wt%, the HTHP volume drops below 20 mL. It indicates that the comb-shaped copolymer can effectively control the filtrate loss volume at high temperatures. Adsorption groups such as the amide group can be anchored to the clay particles through hydrogen bonds. It allows for the long side chains on the comb-shaped structure to sterically hinder clay particle collision and allows for hydration groups such as SO_3_^−^ to increase the thickness of the electrical double layer. The addition of the copolymer helps reduce clay particle aggregation under high temperature and maintains colloidal stability by imparting the steric hindrance and electrostatic repulsion. The clay particle size distribution is therefore modified and helps form a thin and less permeable filter cake to control filtration.

**Fig. 4:**
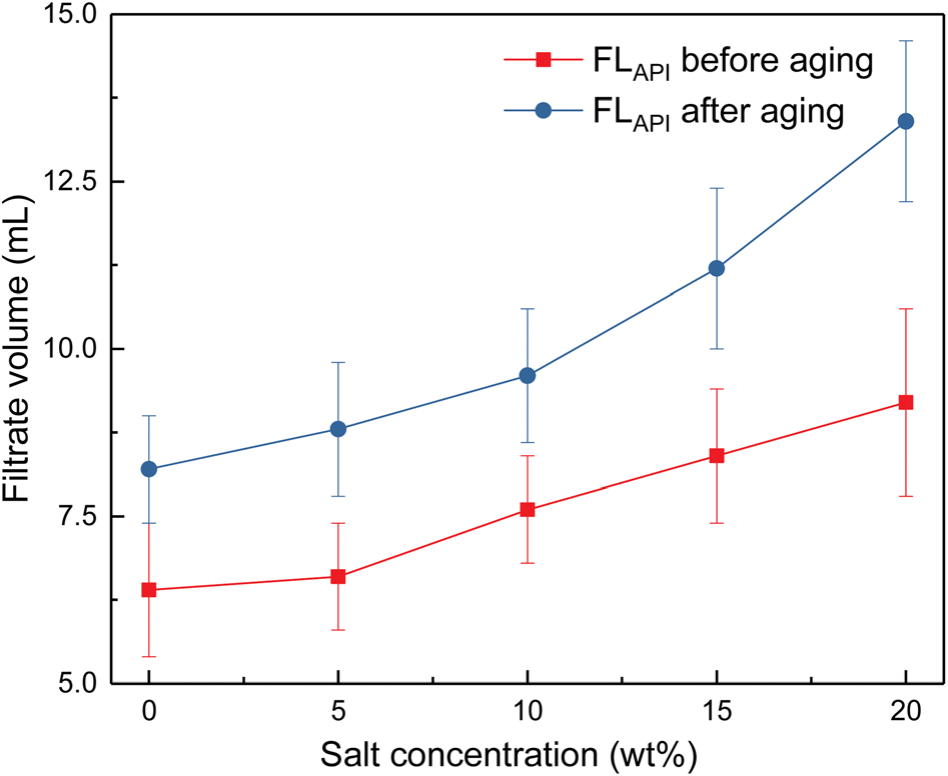
Impact of salt concentration on filtrate loss volume of drilling fluid loaded with 2 wt% copolymer. API filtration volume before aging (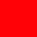) and after aging at 180 °C (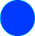).

**Fig. 5:**
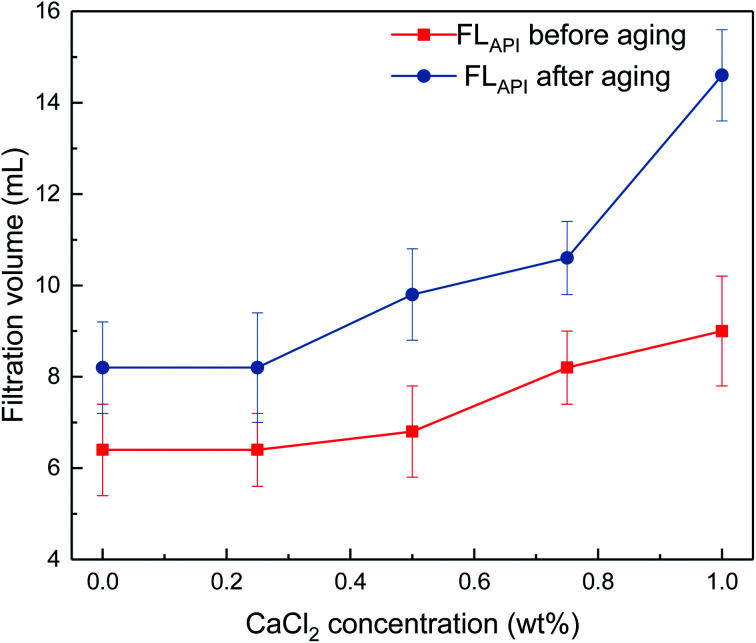
Impact of calcium chloride concentration on filtrate loss volume of drilling fluid loaded with 2 wt% copolymer. API filtration volume before aging (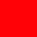) and after aging at 180 °C (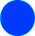).

The effects of sodium chloride and calcium chloride on the filtration properties were investigated. The results are shown in Fig. 4 and Fig. 5.

It can be seen from Fig. 4 and 5 that the filtrate loss volume increases with salinity in the drilling fluid, and the upward trend worsens with increasing temperature. The salt-screening effect can reduce the electrical double layer of clay particles, resulting in aggregation between particles. However, the filtrate loss volumes are less than 15 mL after aging at high salinity, and no severe loss occurs at saturated-salt and calcium contamination. The comb-shaped structure can screen the effect of salinity on the clay particles, protect the clay particles from aggregation and help improve their colloidal stability. Reasonable clay particle size distribution is maintained and a high-quality filter cake is formed to reduce the filtration volume at high temperatures and high salinity.

### 3.3 Comparison with commercial counterparts

#### 3.3.1 Drilling fluid performance

Given that the comb-shaped copolymer can reduce filtration in a high-salinity and high-temperature environment, three other prevailing counterparts are selected for comparison. The mixture of sulfonated phenolic resin (SMP) and sulfonated lignite (SMC) are often used to control filtration in high-temperature deep wells with temperatures up to 220 °C. The polymeric product PAC (through copolymerization of acrylic acid, acrylamide, acrylonitrile and sodium allylsulfonate) is a high-temperature filtrate loss reducer that normally increases the drilling fluid viscosity. The filtrate loss reducer NS-1 can be used for an environment at a temperature of 180 °C.^35^ The rheological properties and filtration control ability of each product is shown in Table 5.

**Table 5:** Drilling fluid properties containing different filtrate loss reducers before and after the aging process

Test samples	AV (mPa s)		PV (mPa s)		YP (Pa)		FL_API_ (mL)	
	Before aging	After aging	Before aging	After aging	Before aging	After aging	Before aging	After aging
1 wt% comb-shaped copolymer^a^	17.0	12.5	12	9	5.0	3.5	7.8	9.4
1 wt% NS-1^a^	16.5	13.0	11	9	4.5	4.0	9.4	19.2
0.4 wt% PAC^a^	26.5	20.5	21	18	5.5	3.0	10.8	17.4
3% SMP^b^	5.5	7.5	5	6	0.5	1.5	23	45.0
3% SMC^b^	8.5	5.5	7	4.5	2.5	1.0	16.8	30.0
3% SMP + 3% SMC^a^	9.5	28.0	8	20	1.5	13.0	4.2	7.4

a 200 °C aging process.

b 120 °C aging process.

According to Table 5, the comb-shaped copolymer has a lower filtrate loss volume compared to NS-1 at high temperature. Due to intramolecular and/or intermolecular associations by hydrogen bonds in the comb-shaped structure, networks are built in the clay dispersion, which help reduce filter cake permeability. Meanwhile, the comb-shaped copolymer has a higher contribution of ring structures to improve the copolymer temperature resistance and possesses more anchoring groups to facilitate adsorption. Therefore, the comb-shaped copolymer structure increases the temperature resistance compared to NS-1 with similar monomers and improves the colloidal stability by incorporating long side chains.

Another polymeric filtrate loss reducer PAC can viscosify the drilling fluid even in low doses. PAC normally has a high molecular weight and has a linear structure, which inherently increases flow resistance. The filtrate loss volume of PAC is higher than that of the comb-shaped copolymer, possibly because the linear structure is less effective in covering and sealing the pores of the filter cake. The comb-shaped copolymer will not viscosify the drilling fluid like linear PAC by maintaining a suitably distributed and well-dispersed clay suspension through steric and electric hindrance.

Sulfonated phenolic resin (SMP) and sulfonated lignite (SMC) are often used together to control filtration under high temperature (200 °C). According to Table 5, either one used alone has a high filtrate loss volume at 120 °C, possibly because they are weakly crosslinked. However, the mixture of SMP and SMC shows excellent filtrate loss control ability in a 1 : 1 ratio. The HTHP filtration volume is only 7.4 mL after aging at 200 °C. The viscosity increases after aging due to the cross-linking at high temperature. However, the uncontrollable cross-linking structure at high temperature may be continuous and may catastrophically damage the drilling fluid properties. The whole system deteriorates or even solidifies, causing severe damage to the drilling fluid system and affecting drilling operations. The filtrate loss volume of the comb-shaped copolymer laden drilling fluid at high temperature is comparable to that of SMP blends but without the risk of excessive cross-linking.

#### 3.3.2 Filtrate loss reducer adsorption on clay particles

**Fig. 6:**
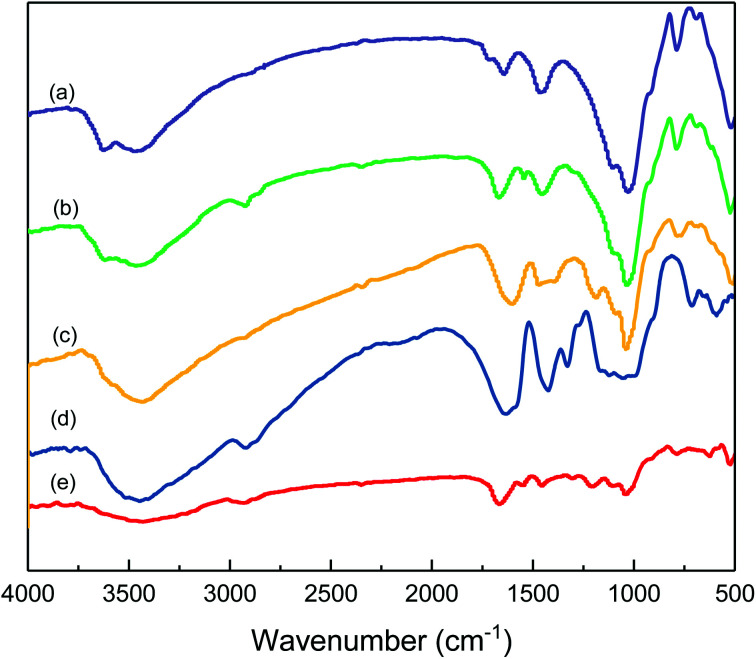
FTIR spectra of the adsorption of hydrated clay and filtrate loss reducers: (a) 4 wt% hydrated clay; (b) 4 wt% hydrated clay treated with 2 wt% comb-shaped copolymer reducer; (c) 4 wt% hydrated clay treated with a mixture of 3 wt% SMP and 3 wt% SMC; (d) 4 wt% hydrated clay treated with 0.3 wt% PAC; (e) 4 wt% hydrated clay treated with 2 wt% NS-1 filtrate loss reducer.

The efficient adsorption between filtrate loss reducers and clay particles is the foundation for best utility. It has been possible to study the association between clay and filtrate loss reducers through the FTIR spectra.^36^ The spectra of the adsorbed species are presented in Fig. 6.

Fig. 6 shows the association of clay particles treated with different filtrate loss reducers. Curve (a) is the typical spectrum for clay. The absorption peak at 3624.03 cm^−1^ (O–H stretching band) is assigned to the Al–O–H structure. The absorption peak at 3458.14 cm^−1^ and 1631.78 cm^−1^ (O–H stretching band and bending band) correspond to water molecules between layers. The strong band at 1027.9 cm^−1^ (Si–O stretching band) and 520.15 cm^−1^ (Si–O bending band) suggest the typical clay spectrum. For the sample of clay treated with the comb-shaped copolymer in curve (b), the absorption peaks at 2921.70 cm^−1^ and 2858.14 cm^−1^ (–C–H stretching band) and the peaks at 1676.74 cm^−1^ and 1548.84 cm^−1^ (amide group) suggest that the adsorption of the copolymer and clay particles is through a hydrogen bond. For samples treated with SMP and SMC in curve (c), the peak at 1186.04 cm^−1^ and 1037.21 cm^−1^ (SO_3_^−^ group) and 1610.80 cm^−1^ (C–C stretching bond) suggest the adsorption of SMP on clay particles. Other filtrate loss reducers such as PAC and NS-1 also show adsorption on clay particles with functional groups present in the spectra. All the filtrate loss reducers possess large number of –OH groups, except for NS-1, presumably because NS-1 has fewer hydration groups.

#### 3.3.3 Self-assembly of filtrate loss reducers

**Fig. 7:**
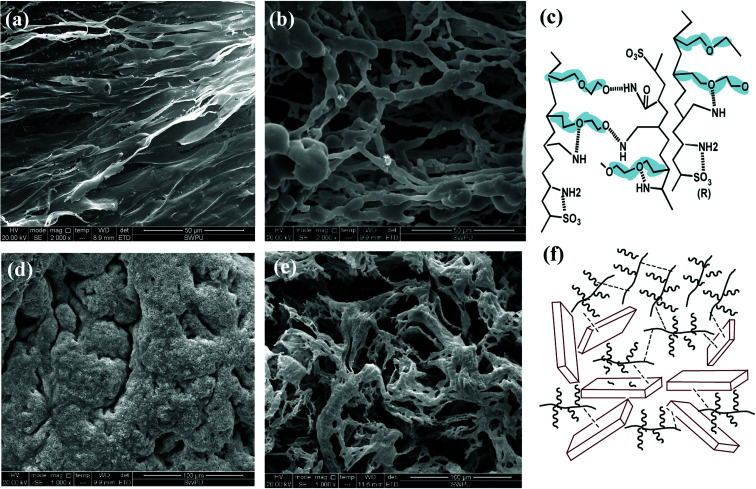
Self-assembly of 1 wt% comb-shaped copolymer and the association with clay particles (a) before aging (2000×) and (b) after aging at 200 °C (2000×). SEM image of 10 wt% salt in comb-shaped copolymer solution with clay particles (d) before aging (1000×) and (e) after aging at 180 °C (2000×). Conceptual illustration of comb-shaped copolymer (c) and association with clay particles (f).

To further probe the working mechanisms of different filtrate loss reducers for water-based drilling fluids, SEM was employed to observe the morphology of each product in the aqueous solution with respect to clay particles adsorption and the impact of salinity on the morphological image, as shown in Fig. 7.

The SEM image in Fig. 7(a) shows that the self-assembly of the copolymer is horizontal. The compact 3-D film structure is mainly for intramolecular and intermolecular association between functional groups in the side chains through hydrogen bonding. The considerable amount of ether oxygen in the side chains provide large amounts of hydrogen bonds association. Numerous copolymer molecules are connected through hydrogen bonds between the hydrogen atom in the amide group and the oxygen atom in the ether oxygen and sulfonic acid group (Fig. 7(c)). The formed film presents a smooth and stretched image because hydrogen bonds are mainly negative, as the N atom in the amide group has a weak attraction to H atoms, resulting in weak electrical repulsion between film structures. Meanwhile, the long side chains endow the main chains of the comb-shaped structure with rigidity and the copolymers present a smooth and regular pattern in solution. The compact network helps reduce filter cake permeability and corresponds to the result of low filtrate volume (Fig. 3). The compact film explains that the addition of the copolymer into the clay suspension can increase the viscosity slightly as more strength is required to break the structure.

As shown in Fig. 7(b), partial film structures are broken into small crossline networks after aging. The long side chains enhance the rigidity of the main chains by providing steric barriers, reducing the effect of temperature on the copolymer. The other stretched and connected networks are able to prevent the flocculation of clay particles. No large clay aggregates occur after hot rolling. The colloidal dispersions are maintained and particle size distribution of clay particles are ideal for a compact pile-up in the mud cake. The remaining structure can effectively fill in the mud cake holes and reduce its permeability, which agrees with the relative low filtrate loss volume (FL_API_ < 10 mL) in the comb-shaped copolymer-laden drilling fluids after aging.

Fig. 7(c) suggests that the increased salinity leads to flat films that need to be curled and folded. Because Na^+^ attracts both anions and negative hydrogen bonds, electrical repulsion between film structures is reduced and molecules become curled or twisted. However, clay particles are well adsorbed and packaged by the copolymer films as a result of the heightened adsorptive capacity of the comb-shaped structure and clay particles (Fig. 7(f)). Hydrated shells of the clay particles are maintained with the help of ionic groups in a high-salinity environment. Fig. 7(d) shows that the curly slice of the films is broken into connected small curly debris at high temperature and salinity. No clay particles aggregate under the protection of the copolymer. Clay particles can be adsorbed onto films and can be packaged by copolymer films. Therefore, partial copolymer networks help maintain a low filtrate loss volume and work well at high-temperature and -salinity conditions.

**Fig. 8:**
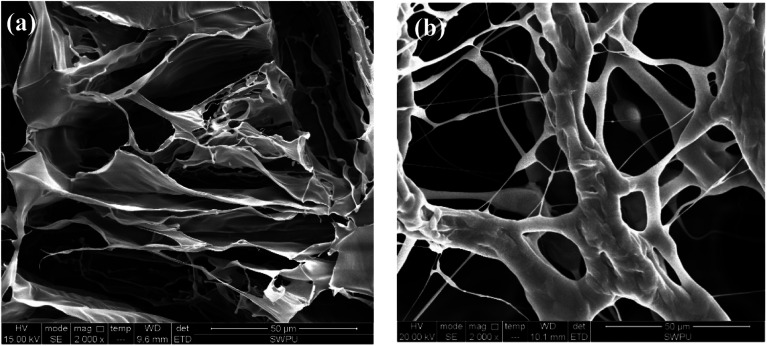
Self-assembly of 1 wt% filtrate loss reducer NS-1 with clay particles (a) before aging (2000×) and (b) after aging at 180 °C (2000×).

The similar polymeric filtrate loss reducer NS-1 with a linear structure was prepared for comparison.^35^ The SEM of NS-1 before and after aging at 180 °C is shown in Fig. 8.

Only large areas of the thin flaky films are found in the SEM image of NS-1 in aqueous solution (Fig. 8(a)). The formed films are quite sparse and less dense due to a simple linear structure. Without long side chains, the intramolecular and intermolecular association is weaker due to lack of sufficient hydrogen bonding points. Therefore, the irregular self-assembly of NS-1 is less compact and not complexly connected compared with that of the comb-shaped copolymer. The sparse films in NS-1 are not effective to reduce filter cake permeability, so that the filtration volume of NS-1 is higher than that of the comb-shaped copolymer.

Similar thermal degradation of the NS-1 is found in Fig. 8(b). Large areas of flaky films degrade into a small chain structure. The linear structure is more likely to thermally degrade and has lower temperature tolerance, presumably on account of lacking long side chains to sterically improve the main chain rigidity. Partial hydration groups in NS-1 break off from the main chains to create hydrophobic chains. The thermal degradation ability of linear NS-1 is lower than that of the comb-shaped copolymer.

**Fig. 9:**
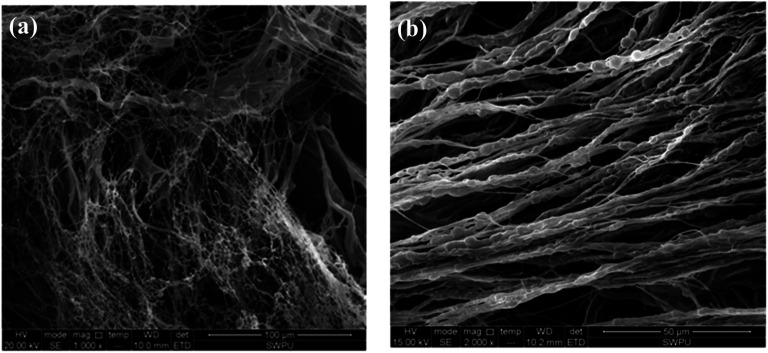
Self-assembly of PAC solutions at different concentrations: (a) 0.2 wt% (1000×); (b) 1 wt% (2000×).^37^

Another example of polymeric product PAC with a linear structure is selected for comparison. It is commonly used at 160–180 °C and often causes the drilling fluid to have a high viscosity. The SEM of the PAC solution is shown in Fig. 9.^37^

As shown in Fig. 9, an irregular and separated filiform structure is formed with the PAC solution at low concentrations (0.2 wt%). When the concentration is increased to 1 wt%, coarser lines are formed, aligned in a single direction. Because the chemical structure of PAC is mostly linear, no intertwined networks are formed in this copolymer, and the lines are separated from each other. Due to the lack of intramolecular and intermolecular association induced by functional groups in the side chains, molecules in PAC are less connected with each other and the morphological images of PAC cannot form compact film structures like the comb-shaped structure. As a result, the filtration volume of PAC is higher than that of the comb-shaped copolymer. Moreover, the results consistent with the results shown in Table 5 that PAC causes the drilling fluid to have high viscosity even in low doses. Separated coarser lines lead to difficulty in the flow of the drilling fluid and make them more difficult to break down at shear stress.

**Scheme 2:**
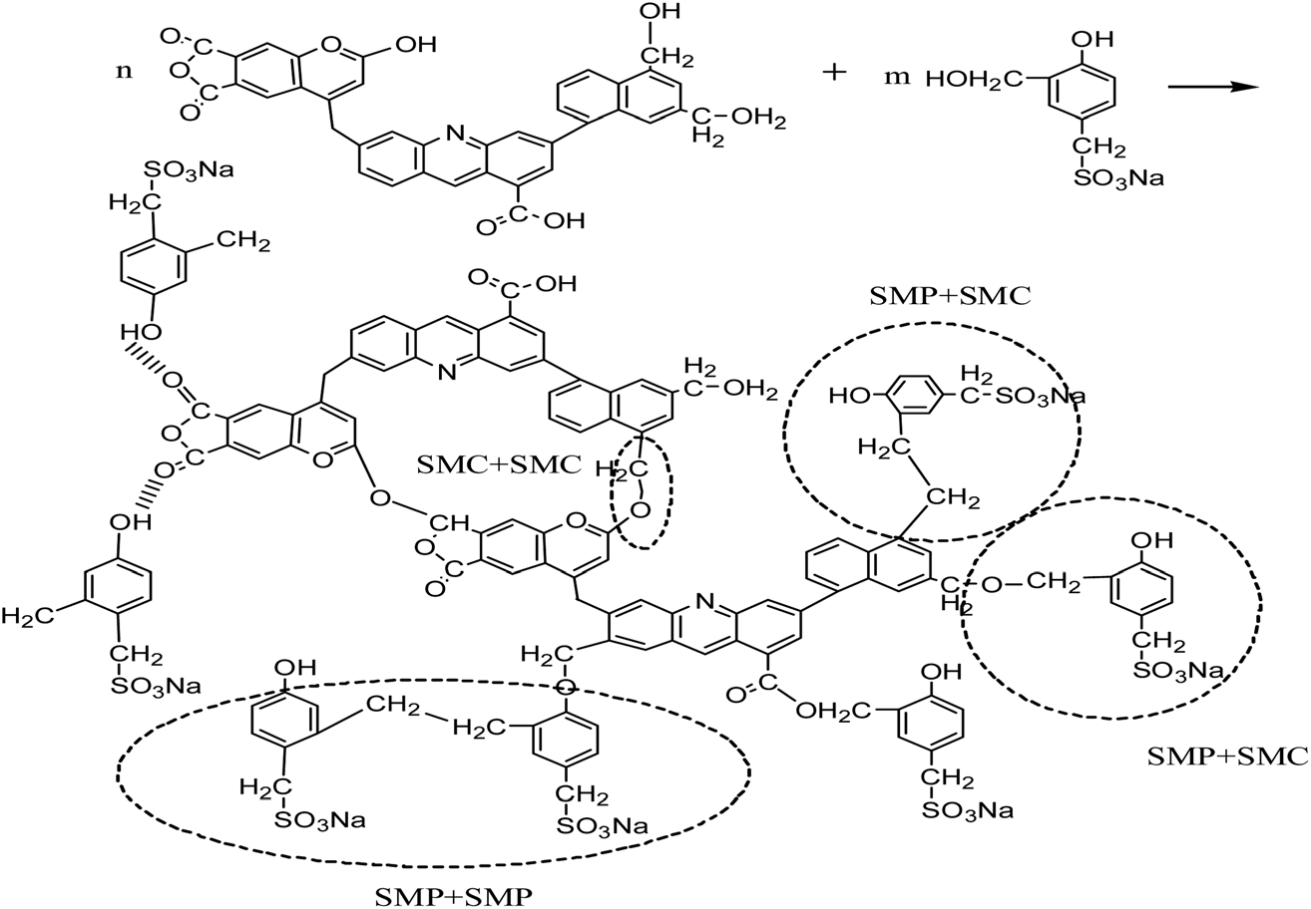
Proposed cross-linking mechanism between SMP and SMC.

We also selected high temperature resistant filtrate loss reducers SMP and SMC blends for comparison, which are normally used in the deep wells up to 220 °C. The proposed cross-linking mechanism between SMP and SMC^38–40^ is shown in Scheme 2.

**Fig. 10:**
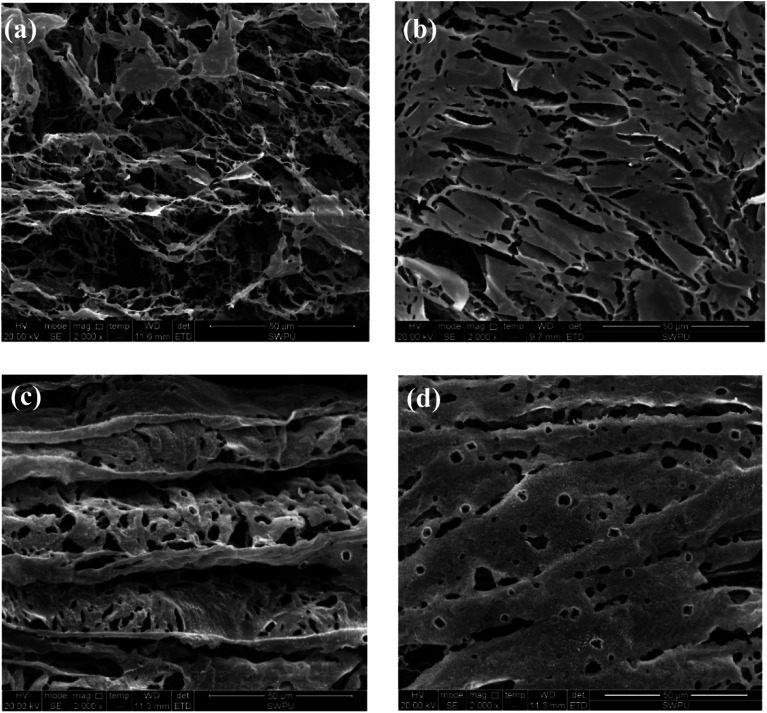
Self-assembly of 3 wt% SMP and 3 wt% SMC compound in aqueous solution and its association with clay particles. The SEM image of SMP and SMC in aqueous solution (a) before aging (2000×) and (b) after aging at 200 °C (2000×). Self-assembly of association between clay particles and SMP and SMC compounds with 10 wt% salt (c) before aging (2000×) and (d) after aging at 200 °C (2000×).

At high temperature, SMP molecules can react with each other through intramolecular dehydration between hydroxymethyl and reactive hydration in the benzene ring to form methylene. The structure of SMC has not been fully identified so far. It is generally believed that basic structures of SMC are aromatic rings connected with sulfur ether, methyl, methyl ether and ether, while being surrounded by amino acids, amino sugars, numerous hydroxyls and carboxyls. The formation of hydrogen bonds among SMC molecules. The SMP and SMC molecules can be cross-linked, as the phenolic hydroxyl group in the SMP molecules react with methoxy or carbonyl in the SMC molecules^41^ or through π–π stacking of aromatic rings^42^ or other intermolecular forces. We used SEM to characterize the crosslinking structure after hot rolling, as shown in Fig. 10.

As can be seen from Fig. 10(a), an irregular line-to-line structure is formed when SMP and SMC molecules are weakly connected before aging. The formed structure is loose, so that the viscosity of the drilling fluid before aging is low. However, the self-assembly of SMP and SMC after aging (Fig. 10(b)) is found with compact and structured films, extending horizontally and vertically in the aqueous solution. The strong crosslinking behavior of the SMP and SMC molecules is observed after hot rolling. Multiple cross-linking behaviours—within the SMP molecules, between SMP molecule and SMC molecule, and within SMC molecules—are conducive to a compact dimensional structure after hot rolling. It is evident from Table 5 that only the mixture of SMP and SMC can effectively control the filtrate loss at high temperature; we therefore conclude that the compact film structure is mainly driven by the cross-linking behaviour between SMP and SMC molecules, which helps control the filtration at high temperature. Clay particles are enveloped within films and protected from aggregation under high temperature. It is possible to form a vertical film to increase the strength and deformability and hence improve the filter cake quality.

The self-assembly in Fig. 10(c) suggests that salt addition can also lead to curling of the film. The salt-screening effect compensates for anions in the mixture and reduces the electrical repulsion between molecules. Then the films are curled and folded. After hot rolling as shown in Fig. 10(d), the cross-linking between SMP and SMC molecules occurs simultaneously without disruption of the salt. A large area of well-ordered films is formed due to the chemical reaction between hydroxymethyl in SMP and functional groups in SMC at high temperature. It explains why SMP and SMC can effectively control the filtration into formation at high salinity and high temperature. However, the drawback of cross-linking behavior is that it is quite difficult to control and handle at high temperatures and an overly crosslinked structure can severely jeopardize the colloidal system.

## 4 Conclusions

This work has synthesized a comb-shaped copolymer using AMPS, APEG, NVP and SSS monomers through aqueous free-radical copolymerization for application as filtrate loss reducers in water-based drilling fluids. FTIR results confirm the chemical structure of the as-prepared copolymer. TG-DSC suggests that the thermal degradation of the copolymer is not obvious until 295.24 °C. The comb-shaped structure helps maintain the rheological properties of the drilling fluid in high-temperature and high-salinity environments, which results from the long PEG chains sterically stabilizing the colloids by protruding into the suspension. The filtrate loss volumes are less than 15 mL at high salinity after aging.

The filtrate loss volume of the comb-shaped copolymer-laden drilling fluid at high temperature is comparable to that of the SMP blends but without the risk of the excessive cross-linking behavior. The comb-shaped copolymer structure increases the temperature resistance compared to that of NS-1 with similar monomers. The comb-shaped copolymer does not viscosify the drilling fluid like linear PAC does by maintaining a suitably distributed and well-dispersed clay suspension through steric and electric hindrance.

The self-assembly of the comb-shaped copolymer with a compact 3-D stretched film structure is possible because PEG chains provide plenty of ether oxygen in the long side chains for hydrogen bonding points that enable intramolecular and intermolecular association. Though retained with small curly debris at high temperature and salts, the remaining copolymer networks can still reduce filtration effectively. The formed films in NS-1 are quite sparse and less dense with weaker intramolecular and intermolecular association. Large areas of flaky films thermally degrade into a small chain structure, suggesting a lower temperature resistance. Only the separated filiform structure and coarse lines are found in polymeric PAC with a linear structure, which increase the viscosity of the drilling fluid. The compact and structure films are found with the mixture of SMP and SMC. Such films are even heightened at high temperature. Large areas of the well-ordered films remain intact with salt, suggesting that the cross-linking reaction between SMP and SMC molecules is not affected by salt addition.

## Conflicts of interest

The authors declare that they have no conflicts of interest.

## Supplementary Material
